# Accuracy of Nodal Positivity in Inadequate Lymphadenectomy in Pancreaticoduodenectomy for Pancreatic Ductal Adenocarcinoma: A Population Study Using the US SEER Database

**DOI:** 10.3389/fonc.2019.01386

**Published:** 2019-12-06

**Authors:** Weishen Wang, Ziyun Shen, Yusheng Shi, Siyi Zou, Ningzhen Fu, Yu Jiang, Zhiwei Xu, Hao Chen, Xiaxing Deng, Baiyong Shen

**Affiliations:** Department of General Surgery, Pancreatic Disease Center, Ruijin Hospital Affiliated to Shanghai Jiaotong University School of Medicine, Shanghai, China

**Keywords:** pancreatic ductal adenocarcinoma (PDAC), pancreaticoduodenectomy (PD), inadequate lymphadenectomy, surveillance epidemiology and end results (SEER), examined lymph nodes

## Abstract

**Objectives:** The optimal number of the examined lymph nodes (ELNs) in pancreaticoduodenectomy for pancreatic ductal adenocarcinoma has been widely studied. However, the accuracy of nodal positivity for the patients with inadequate lymphadenectomy is still unclear. The purpose of our study was to determine the accuracy of the number of positive nodes reported for patients with 1–3 positive nodes and the probability that 4 or more nodes could be positive along with tumor size and number of nodes examined.

**Methods:** We obtained data on patients who underwent pancreaticoduodenectomy for resectable pancreatic ductal adenocarcinoma diagnosed during 2004–2013 from the US Surveillance, Epidemiology, and End Results registry. An mathematical model based on Hypergeometric Distribution and Bayes' Theorem was used to estimate the accuracy.

**Results:** Among the 9,945 patients, 55.6% underwent inadequate lymphadenectomy. Of them, 1,842, 6,049, and 2,054 had T1, T2, and T3 stage disease, respectively. The accuracy of the number of observed positive nodes increased as the number of ELNs increased and the tumor size decreased. To rule out the possibility of N2 stage (4 and more positive nodes), there should be at least 13 ELNs for the patients with 1 observed positive lymph node and 14 for the patients with 2.

**Conclusion:** Inadequate lymphadenectomy could result in underestimation of the N stage, and this would have adverse impact on recurrence, efficacy of postoperative treatment, and even overall survival. This model combined with the observed positive lymph nodes, the number of ELNs, and tumor size could provide a more accurate determination of nodal positivity of these patients.

## Introduction

Pancreatic cancer is the seventh leading cause of cancer death worldwide (1). Surgical resection is the only potentially curative therapy for pancreatic cancer. Pancreaticoduodenectomy is the standard surgery for pancreatic head cancer and is widely performed across countries (2). Lymphadenectomy is an indispensable procedure of pancreaticoduodenectomy for pancreatic cancer. However, despite resection and chemotherapy, patients still have poor prognosis, particularly those with positive lymph nodes in lymphadenectomy (3).

In the past 10 years, considerable attention has been paid to the ELNs, the number of positive lymph nodes (PLNs), and the lymph node ratio (LNR) in pancreatic ductal adenocarcinoma. Some research showed that the higher the number of ELNs or PLNs, the worse the median overall survival (OS) and 5-year OS (4). LNR is directly associated with disease-free survival and OS, making it one of the most powerful prognostic predictors after resection of pancreatic cancers (5–8).

The International Study Group on Pancreatic Surgery (ISGPS) recommended that at least 15 lymph nodes should be detected in pancreaticoduodenectomy to ensure adequate pathologic staging of the disease (9). However, excising fewer local lymph nodes, with the purpose of decreasing subsequent morbidity and mortality, has become a recent trend in surgery (10, 11). Nonetheless, the optimal number of nodes that need to be examined in node-positive patients to accurately determine the number of involved lymph nodes is yet to be determined. Therefore, this study aimed to determine the accuracy of the number of positive nodes reported for patients with 1–3 positive nodes and the probability that 4 or more nodes could be positive along with tumor size and number of nodes examined.

## Methods

### Patients and Data Source

We collected data from the US Surveillance, Epidemiology and End Results (SEER) database. SEER stores data on cancer incidence from population-based cancer registries covering ~34.6% of the US population. Data of patients with pancreatic ductal adenocarcinoma (PDAC) (International Classification of Diseases for Oncology, third edition, ICD-O-3 histology/behavior codes: 8140/3 and 8500/3) who underwent pancreaticoduodenectomy was collected. The patients with missing information in patient demographics, tumor data, perioperative treatment were excluded. All patients had at least 1 lymph node examined. Patients with metastasis of PDAC were excluded. Finally, we analyzed data of 9,945 patients with T1–3 stage who underwent pancreaticoduodenectomy for PDAC of the head of the pancreas between 2004 and 2013 (Supplementary Figure 1).

### Mathematical Model

A mathematical model based on Hypergeometric Distribution and Bayes' Theorem was created to estimate the true number of involved lymph nodes for patients with partial dissections (12, 13). In this model, there is no difference among all involved lymph nodes. The 90% certainty is used to identify the accuracy of the nodal positivity after an inadequate lymphadenectomy in pancreaticoduodenectomy for PDAC. In pancreaticoduodenectomy for PDAC, the average total number of lymph nodes was assumed to be 15, based on the ISGPS (9). Two criteria were used in this model: (1) The probability of having M involved nodes, q(M) where: q(M) = (No. of patients with M involved nodes)/(total number of patients in group). (2) P represents the probability of involved lymph nodes (m) observed in the sample size resected (n). N represents the total number of lymph nodes positive in the lymphadenectomy in pancreaticoduodenectomy for PDAC with M nodes positive. Thus, P(n, m, N, M) was obtained from the common hypergeometric distribution, where:

(1) $$\text{P}(\text{n},\text{m},\text{N},\text{M})=\frac{\left(\begin{array}{c} M\\ m \end{array}\right)\left(\begin{array}{c} N-\;M\\ n-\;m \end{array}\right)}{\left(\begin{array}{c} N\\ n \end{array}\right)}$$

Patients with at least 15 lymph nodes examined in pancreaticoduodenectomy for PDAC should have a complete dissection. We used the data of those patients to find the probability of having involved lymph nodes. These probabilities are based upon observed sampling combinations, and thus followed by Bayes' Theorem. Further, calculations in T1, T2, and T3 were conducted separately. r(M, n, m) was defined as the probability of a patient having M involved nodes, after involved lymph nodes (m) been observed in the sample size resected (n):

(2) $$\begin{array}{r} \text{r}\left(\text{M},\text{n},\text{m}\right)=\frac{q\left(M\right)\times \;P\left(n,\;m,\;N,M\right)}{\sum_{M}q\left(M\right)\times P\left(n,m,N,m\right)} \end{array}$$

The denominator is necessary to ensure that:

(3) $$\begin{array}{r} \underset{M}{\sum }\text{ r}\left(\text{M},\text{ n},\text{ m}\right)=\;1 \end{array}$$

In the TNM staging system, metastasis to ≥4 regional lymph nodes represented N2 stage, which corresponds to stage III, regardless of T Stage, in the 8th American Joint Committee on Cancer (AJCC) TNM guideline (Supplementary Table 1) (14). Thus, this mathematical model was also used to calculate the probabilities of having 4 or more positive nodes, given that a specific sampling combination has been observed.

Note that this calculation entails the summation of r(M,n,m) over M, from 4 to 15.

### Statistical Analysis

ELNs and N0/N1/N2 stage in different T stages were compared using Student's *t*-test. Data were analyzed using IBM SPSS Statistics for Windows version 24.0 (SPSS Inc.). All tests were two-tailed, and *p* < 0.05 was considered statistically significant.

## Results

### Patient Characteristics

**Figure 1:**
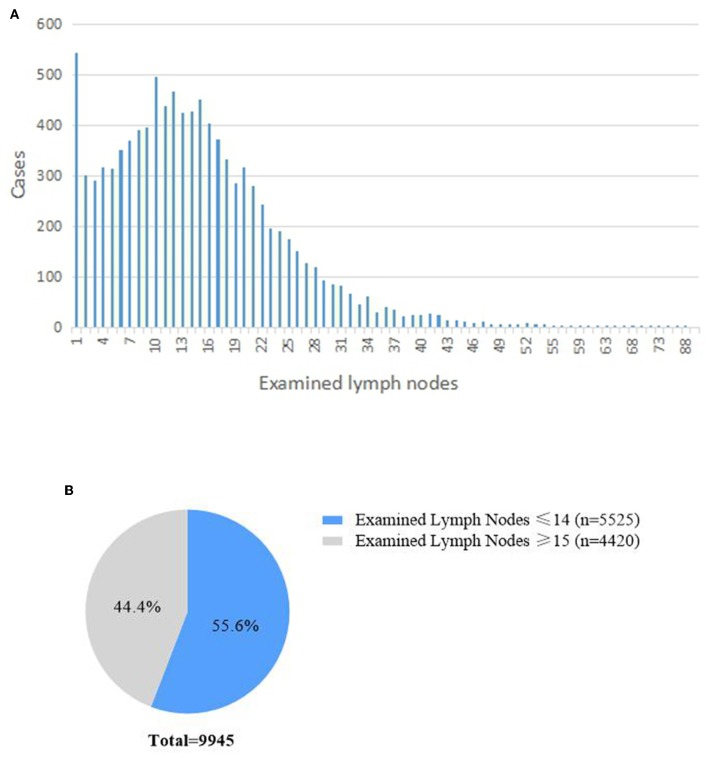
The distributions of the examined lymph nodes.

A total of 9,945 patients registered in the SEER database were included in this study. The median number of ELNs was 13 (7–20). The distributions of the number of ELN are shown in Figures 1A,B. In total, 3,272 (32.9%) had <10 ELNs, 2,253 (22.7%) had 10–14 ELNS, and 4,420 (44.4%) had more than 15 ELNs. There were 5,525 (55.6%) patients who underwent inadequate lymphadenectomy (i.e., <15 ELNs were harvested during pancreaticoduodenectomy).

With respect to T stage according to the 8th AJCC TNM staging system, 1,842 (18.5%), 6,049 (60.8%), and 2,054 (20.7%) had T1, T2, and T3 stage disease, respectively. There were significant differences in ELNs and N0/N1/N2 stage between the T stages (Table 1). The percentage of patients with N2 stage increased as the T stage advanced.

**Table 1:** Lymph node status for different T stages.

**Variables**	**T1**	**T2**	**T3**	
	***n* = 1,842**	***n* = 6,049**	***n* = 2,054**	***P*-value**
ELN^*^	12 (7–19)	13 (8–20)	14 (7–20)	0.000
Node negative^**^	881 (47.8%)	2,010 (33.2%)	645 (31.4%)	0.000
Node positive^***^				0.000
N1	728 (39.5%)	2,592 (42.9%)	818 (39.8%)	
N2	233 (12.7%)	1,447 (23.9%)	591 (28.8%)	

* *P-value, T1 vs. T2 (0.001), T1 vs. T3 (0.001), T2 vs. T3 (0.498)*.

** *P-value, T1 vs. T2 (0.000), T1 vs. T3 (0.000), T2 vs. T3 (0.128)*.

*** *P-value, T1 vs. T2 (0.000), T1 vs. T3 (0.000), T2 vs. T3 (0.000)*.

### Accuracy of the Number of Positive Lymph Nodes Reported

The accuracy of the number of PLNs reported for patients who undergone pancreaticoduodenectomy with T1 stage is shown in Table 2. For example, 14 lymph nodes were examined in a patient with T1 tumor, and the accuracy of observation of 1 positive lymph node was 91.1%; 2 positive nodes, 84.8%; and 3 positive nodes, 82.0%.

**Table 2:** Accuracy of the number of positive lymph nodes reported according to the number of examined lymph nodes in patients with T1 tumor.

	**Number of positive nodes observed**		
**ELN**	**1**	**2**	**3**
4	21.9%	8.2%	2.8%
5	26.9%	11.7%	4.9%
6	32.3%	15.8%	7.9%
7	38.2%	20.7%	12.0%
8	44.4%	26.4%	17.2%
9	51.2%	33.1%	23.6%
10	58.4%	40.8%	31.4%
11	66.0%	49.7%	40.9%
12	74.0%	59.9%	52.4%
13	82.4%	71.5%	66.1%
14	91.1%	84.8%	82.0%

The accuracy of the number of positive nodes reported improved as the number of ELNs increased. Meanwhile, if more positive nodes were observed, more lymph nodes should be harvested to increase the accuracy of nodal positivity assessment. For example, for an 80% accuracy of 1 positive lymph node, the minimal number of ELNs was 13; however, for 2 or more positive nodes, 14 lymph nodes should be examined to reach the same accuracy.

Tables 3, 4 shows the accuracy of PLNs reported in patients with T2 and T3 tumors. Note that regardless of the number of PLNs observed, the accuracy decreased as the tumor size increased.

**Table 3:** Accuracy of the number of positive lymph nodes reported according to the number of examined lymph nodes in patients with T2 tumor.

	**Number of positive nodes observed**		
**ELN**	**1**	**2**	**3**
4	16.3%	5.4%	1.5%
5	21.1%	8.4%	3.1%
6	26.3%	12.1%	5.5%
7	32.2%	16.8%	8.8%
8	38.6%	22.3%	13.5%
9	45.6%	29.0%	19.6%
10	53.3%	36.8%	27.4%
11	61.5%	45.9%	37.1%
12	70.3%	56.5%	48.9%
13	79.7%	68.9%	63.2%
14	89.6%	83.3%	80.1%

**Table 4:** Accuracy of the number of positive lymph nodes observed according to the number of examined lymph nodes in patients with T3 tumor.

	**Number of positive nodes observed**		
**ELN**	**1**	**2**	**3**
4	12.9%	4.8%	1.3%
5	17.0%	7.8%	2.8%
6	21.6%	11.5%	5.4%
7	26.8%	16.1%	9.1%
8	32.6%	21.6%	14.3%
9	39.3%	28.1%	21.2%
10	46.8%	35.5%	30.0%
11	55.3%	44.2%	40.6%
12	64.9%	54.5%	52.9%
13	75.6%	66.7%	66.6%
14	87.3%	81.6%	82.2%

### Probability of N2 Stage

In the TNM staging system, metastasis in 4 or more regional lymph nodes corresponded to N2 stage. For patients with 1–3 PLNs, the probability of 4 and more PLNs in different T stages is shown in Tables 5–7. As table 6 the number of ELNs increased, the risk of 4 and more PLNs decreased. To lower the possibility of N2 stage to 0%, the minimal number of ELNs was 13 and 14 for the patients with 1 and 2 positive lymph nodes, respectively. Meanwhile, for the patients with 3 positive nodes observed, the possibility of N2 stage was equal to the possibility of inaccuracy of 3 PLNs.

**Table 5:** Probability of 4 or more positive lymph nodes in patients with T1 tumor.

	**Number of positive nodes observed**		
**ELN**	**1**	**2**	**3**
4	33.1%	75.7%	97.2%
5	25.3%	67.4%	95.1%
6	18.6%	58.6%	92.1%
7	13.1%	49.5%	88.0%
8	8.6%	40.3%	82.8%
9	5.1%	31.3%	76.4%
10	2.7%	22.6%	68.6%
11	1.1%	14.6%	59.1%
12	0.3%	7.9%	47.6%
13	0.0%	2.8%	33.9%
14	0.0%	0.0%	18.0%

**Table 6:** Probability of 4 or more positive lymph nodes in patients with T2 tumor.

	**Number of positive nodes observed**		
**ELN**	**1**	**2**	**3**
4	42.1%	82.6%	98.5%
5	32.7%	74.8%	96.9%
6	24.3%	65.9%	94.5%
7	17.2%	56.3%	91.2%
8	11.4%	46.3%	86.5%
9	6.9%	36.2%	80.4%
10	3.6%	26.4%	72.6%
11	1.5%	17.3%	62.9%
12	0.4%	9.4%	51.1%
13	0.0%	3.4%	36.8%
14	0.0%	0.0%	19.9%

**Table 7:** Probability of 4 or more positive lymph nodes in patients with T3 tumor.

	**Number of positive nodes observed**		
**ELN**	**1**	**2**	**3**
4	43.3%	83.3%	98.7%
5	33.4%	74.7%	97.2%
6	24.8%	65.1%	94.6%
7	17.6%	54.8%	90.9%
8	11.8%	44.3%	85.7%
9	7.2%	34.1%	78.8%
10	3.9%	24.5%	70.0%
11	1.7%	16.0%	59.4%
12	0.5%	8.8%	47.1%
13	0.0%	3.3%	33.4%
14	0.0%	0.0%	17.8%

## Discussion

The number of PLNs is associated with the survival of patients with pancreatic cancer (3). The ISGPS consensus statement (9) recommends at least 15 lymph nodes to be retrieved during pancreaticoduodenectomy. However, data from SEER showed that more than 50% of the patients undergoing pancreaticoduodenectomy received inadequate lymphadenectomy. The accuracy of PLNs from these inadequate lymphadenectomies remains unknown. Thus, we used a model by Iyer et al. (12) to predict whether nodal positivity was accurately reported for these patients.

This model provided the accuracy of the number of positive lymph nodes reported from each level of ELNs for the patients with T1–3 tumor. The results indicated that more lymph nodes should be involved to avoid understaging the N stage. However, the minimal number of lymph nodes needed to provide optimal staging could not be summarized from this study. In previous studies, the optimal minimal number of lymph nodes ranged from 11 to 18 (15–18). Huebner et al. (15) suggested that adequate staging of pancreatic cancer required more than 11 lymph nodes; however, based on our model, we found that this recommendation only had a maximum predictive accuracy of 66.0%. Valsangkar et al. (16) proposed an optimal number of 13 based on the SEER database (data from 1973 to 2009), but in our model, this only had an 82.4% accuracy. This indicates that these are not the optimal number of ELNs. It seemed that the N stage was easily overestimated when the ELNs was low. The results from this study indicate that to accurately determine the number of PLNs, there should be at least 15 ELNs. Meanwhile, Arrington et al. (18) suggested 18 ELNs to capture 90% of cases with PLNs. Eskander et al. (17) discussed the evolution of lymph node dissection during 2-year intervals from 2004 to 2012 and reported that the number of ELNs had increased, and more patients were classified as node positive due to the standard lymphadenectomy.

“The more the better” might be a major principle for the lymphadenectomy in the pancreaticoduodenectomy. The results from this study indicated that the more ELNs improved the accuracy of positive nodes and N staging. Warschkow et al. (19) reported similar findings that a higher number of regional lymph nodes retrieved increased not only the accuracy of diagnosis of node-positive pancreatic cancer, but also improved survival in pancreatic cancer. However, there is also a maximum limit on the optimal number of ELNs. Eskander et al. (17) confirmed that no increased benefit was achieved beyond 30 nodes.

We then investigated whether an adequate number of lymph nodes can be obtained from a standard lymphadenectomy. Recent randomized clinical trials (20–24) showed a significant higher number of ELNs in extended lymphadenectomy than in standard lymphadenectomy. However, extended lymphadenectomy did not have any benefits on OS. The median number of ELNs in standard lymphadenectomy ranged from 13 to 17. Extended lymphadenectomy might be better to increase the number of harvested lymph nodes, while standard lymphadenectomy is easier to perform. Lidsky et al. (25) found that compared with patients in low-volume centers, those in high-volume centers tended to have a higher number of ELNs. Therefore, our model is applicable for evaluating outcomes in low-volume centers. However, it should be noted that the harvested number lymph nodes could still be less than the optimal minimal number even standard lymphadenectomy.

Patients with different ELNs, but similar positive lymph nodes could not be distinguished using the TNM classification. Because the number of ELNs is not considered in N staging, LNR was used to evaluate the survival of these patients. Berger et al. (26) first indicated that LNR significantly influenced survival and could thus be used as a prognostic biomarker for pancreatic malignant tumors. This has since been supported by several studies (8, 27, 28). However, LNR has its own limitations. Interestingly, a patient with 1 positive lymph node out of 5 ELNs had the same LNR as a patient with 2 positive lymph nodes out of 10 ELNs. Furthermore, these two patients had the same N stage. The model in the current study could separate these patients according to nodal involvement and could thus be a novel method for a more accurate N staging in pancreatic cancer.

Some limitations in this study warrant emphasis. Patients who had neoadjuvant chemoradiation therapy were not included in this study. Fewer PLNs might be found in these patients, of which the accuracy of nodal positivity needs further evaluation. Moreover, the accuracy of the number of PLNs reported from 15 and more ELNs remains unknown. Lastly, as mentioned above, the minimal number of ELNs for optimal staging could not be determined.

In conclusion, inadequate lymphadenectomy could result in underestimation of the N stage. Our novel model for determining the accuracy of PLNs could provide a more accurate determination of nodal positivity in patients with pancreatic cancer.

## Data Availability Statement

Publicly available datasets were analyzed in this study. This data can be found here: SEER database.

## Author Contributions

WW, ZS, and YS: study conception, design, and drafting of the manuscript. WW, SZ, NF, YJ, and ZX: acquisition of data. WW, ZS, YS, NF, YJ, and HC: analysis and interpretation of data. XD and BS: critical revision.

### Conflict of Interest

The authors declare that the research was conducted in the absence of any commercial or financial relationships that could be construed as a potential conflict of interest.

## Supplementary Material

Supplementary Figure 1 Flow chart.

Supplementary Table 1 The AJCC 8th TNM staging system.

## References

[1] Bray F Ferlay J Soerjomataram I Siegel RL Torre LA Jemal A. Global cancer statistics 2018: GLOBOCAN estimates of incidence and mortality worldwide for 36 cancers in 185 countries. CA Cancer J Clin. (2018) 68:394–424. 10.3322/caac.21492 30207593

[2] Tempero MA Malafa MP Chiorean EG Czito B Scaife C Narang AK. Pancreatic adenocarcinoma, version 1.2019. J Natl Compr Canc Netw. (2019) 17:202–10. 10.6004/jnccn.2019.0003 30865919

[3] Elshaer M Gravante G Kosmin M Riaz A Al-Bahrani A. A systematic review of the prognostic value of lymph node ratio, number of positive nodes and total nodes examined in pancreatic ductal adenocarcinoma. Ann R Coll Surg Engl. (2017) 99:101–6. 10.1308/rcsann.2016.0340 27869496 PMC5392844

[4] Lahat G Lubezky N Gerstenhaber F Nizri E Gysi M Rozenek M. Number of evaluated lymph nodes and positive lymph nodes, lymph node ratio, and log odds evaluation in early-stage pancreatic ductal adenocarcinoma: numerology or valid indicators of patient outcome? World J Surg Oncol. (2016) 14:254. 10.1186/s12957-016-0983-5 27687517 PMC5041551

[5] Morales-Oyarvide V Rubinson DA Dunne RF Kozak MM Bui JL. Lymph node metastases in resected pancreatic ductal adenocarcinoma: predictors of disease recurrence and survival. Br J Cancer. (2017) 117:1874–82. 10.1038/bjc.2017.349 28982112 PMC5729468

[6] Riediger H Keck T Wellner U zur Hausen A Adam U Hopt UT. The lymph node ratio is the strongest prognostic factor after resection of pancreatic cancer. J Gastrointest Surg. (2009) 13:1337–44. 10.1007/s11605-009-0919-2 19418101

[7] Shamseddine AI Mukherji D Melki C Elias E Eloubeidi M Dimassi H. Lymph node ratio is an independent prognostic factor after resection of periampullary malignancies: data from a tertiary referral center in the middle East. Am J Clin Oncol. (2014) 37:13–8. 10.1097/COC.0b013e31826b9c74 23111358

[8] Pawlik TM Gleisner AL Cameron JL Winter JM Assumpcao L Lillemoe KD. Prognostic relevance of lymph node ratio following pancreaticoduodenectomy for pancreatic cancer. Surgery. (2007) 141:610–8. 10.1016/j.surg.2006.12.013 17462460

[9] Tol JA Gouma DJ Bassi C Dervenis C Montorsi M Adham M. Definition of a standard lymphadenectomy in surgery for pancreatic ductal adenocarcinoma: a consensus statement by the International Study Group on Pancreatic Surgery (ISGPS). Surgery. (2014) 156:591–600. 10.1016/j.surg.2014.06.016 25061003 PMC7120678

[10] Michalski CW Kleeff J Wente MN Diener MK Buchler MW Friess H. Systematic review and meta-analysis of standard and extended lymphadenectomy in pancreaticoduodenectomy for pancreatic cancer. Br J Surg. (2007) 94:265–73. 10.1002/bjs.5716 17318801

[11] Iqbal N Lovegrove RE Tilney HS Abraham AT Bhattacharya S Tekkis PP. A comparison of pancreaticoduodenectomy with extended pancreaticoduodenectomy: a meta-analysis of 1909 patients. Eur J Surg Oncol. (2009) 35:79–86. 10.1016/j.ejso.2008.01.002 18356005

[12] Iyer RV Hanlon A Fowble B Freedman G Nicolaou N Anderson P. Accuracy of the extent of axillary nodal positivity related to primary tumor size, number of involved nodes, and number of nodes examined. Int J Radiat Oncol Biol Phys. (2000) 47:1177–83. 10.1016/S0360-3016(00)00574-5 10889370

[13] Kiricuta CI Tausch J. A mathematical model of axillary lymph node involvement based on 1446 complete axillary dissections in patients with breast carcinoma. Cancer. (1992) 69:2496–501. 10.1002/1097-0142(19920515)69:10<2496::AID-CNCR2820691018>3.0.CO;2-T 1568171

[14] Allen PJ Kuk D Castillo CF Basturk O Wolfgang CL Cameron JL. Multi-institutional validation study of the american joint commission on cancer (8th Edition) changes for T and N staging in patients with pancreatic adenocarcinoma. Ann Surg. (2017) 265:185–91. 10.1097/SLA.0000000000001763 27163957 PMC5611666

[15] Huebner M Kendrick M Reid-Lombardo KM Que F Therneau T Qin R. Number of lymph nodes evaluated: prognostic value in pancreatic adenocarcinoma. J Gastrointest Surg. (2012) 16:920–6. 10.1007/s11605-012-1853-2 22421988

[16] Valsangkar NP Bush DM Michaelson JS Ferrone CR Wargo JA Lillemoe KD. N0/N1, PNL, or LNR? The effect of lymph node number on accurate survival prediction in pancreatic ductal adenocarcinoma. J Gastrointest Surg. (2013) 17:257–66. 10.1007/s11605-012-1974-7 23229885 PMC3806050

[17] Eskander MF de Geus SW Kasumova GG Ng SC Al-Refaie W Ayata G. Evolution and impact of lymph node dissection during pancreaticoduodenectomy for pancreatic cancer. Surgery. (2017) 161:968–76. 10.1016/j.surg.2016.09.032 27865602

[18] Arrington AK Price ET Golisch K Riall TS. Pancreatic cancer lymph node resection revisited: a novel calculation of number of lymph nodes required. J Am Coll Surg. (2019) 228:662–9. 10.1016/j.jamcollsurg.2018.12.031 30677528

[19] Warschkow R Widmann B Beutner U Marti L Steffen T Schiesser M. The more the better-lower rate of stage migration and better survival in patients with retrieval of 20 or more regional lymph nodes in pancreatic cancer: a population-based propensity score matched and trend SEER analysis. Pancreas. (2017) 46:648–57. 10.1097/MPA.0000000000000784 28196023

[20] Jang JY Kang JS Han Y Heo JS Choi SH Choi DW. Long-term outcomes and recurrence patterns of standard versus extended pancreatectomy for pancreatic head cancer: a multicenter prospective randomized controlled study. J Hepatobiliary Pancreat Sci. (2017) 24:426–33. 10.1002/jhbp.465 28514000

[21] Nimura Y Nagino M Takao S Takada T Miyazaki K Kawarada Y. Standard versus extended lymphadenectomy in radical pancreatoduodenectomy for ductal adenocarcinoma of the head of the pancreas: long-term results of a Japanese multicenter randomized controlled trial. J Hepatobiliary Pancreat Sci. (2012) 19:230–41. 10.1007/s00534-011-0466-6 22038501

[22] Pedrazzoli S DiCarlo V Dionigi R Mosca F Pederzoli P Pasquali C. Standard versus extended lymphadenectomy associated with pancreatoduodenectomy in the surgical treatment of adenocarcinoma of the head of the pancreas: a multicenter, prospective, randomized study. Lymphadenectomy Study Group. Ann Surg. (1998) 228:508–17. 10.1097/00000658-199810000-00007 9790340 PMC1191525

[23] Yeo CJ Cameron JL Sohn TA Coleman J Sauter PK Hruban RH. Pancreaticoduodenectomy with or without extended retroperitoneal lymphadenectomy for periampullary adenocarcinoma: comparison of morbidity and mortality and short-term outcome. Ann Surg. (1999) 229:613–22; discussion: 22–4. 10.1097/00000658-199905000-00003 10235519 PMC1420805

[24] Farnell MB Pearson RK Sarr MG DiMagno EP Burgart LJ Dahl TR. A prospective randomized trial comparing standard pancreatoduodenectomy with pancreatoduodenectomy with extended lymphadenectomy in resectable pancreatic head adenocarcinoma. Surgery. (2005) 138:618–28; discussion: 28–30. 10.1016/j.surg.2005.06.044 16269290

[25] Lidsky ME Sun Z Nussbaum DP Adam MA Speicher PJ Blazer DG 3rd. Going the extra mile: improved survival for pancreatic cancer patients traveling to high-volume centers. Ann Surg. (2017) 266:333–8. 10.1097/SLA.0000000000001924 27429020

[26] Berger AC Watson JC Ross EA Hoffman JP. The metastatic/examined lymph node ratio is an important prognostic factor after pancreaticoduodenectomy for pancreatic adenocarcinoma. Am Surg. (2004) 70:235–40; discussion: 40. 10.1111/j.1600-6143.2004.00358.x 15055847

[27] House MG Gonen M Jarnagin WR D'Angelica M DeMatteo RP Fong Y. Prognostic significance of pathologic nodal status in patients with resected pancreatic cancer. J Gastrointest Surg. (2007) 11:1549–55. 10.1007/s11605-007-0243-7 17786531

[28] Massucco P Ribero D Sgotto E Mellano A Muratore A Capussotti L. Prognostic significance of lymph node metastases in pancreatic head cancer treated with extended lymphadenectomy: not just a matter of numbers. Ann Surg Oncol. (2009) 16:3323–32. 10.1245/s10434-009-0672-5 19777195

