# Supplementary figures and images for: Accuracy of Nodal Positivity in Inadequate Lymphadenectomy in Pancreaticoduodenectomy for Pancreatic Ductal Adenocarcinoma: A Population Study Using the US SEER Database
Source: Front Oncol. 2019 Dec 6;9:1386. doi: 10.3389/fonc.2019.01386 (PMC6909429; doi:10.3389/fonc.2019.01386)

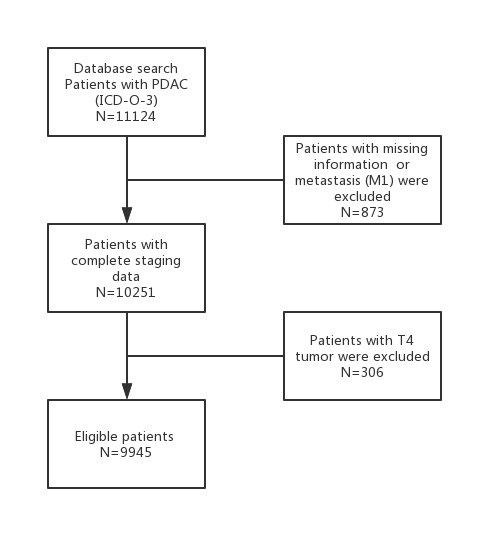

Supplement: Supplementary Figure 1 — Flow chart. [file Image_1.JPEG]
