# Supplementary material for: Accuracy of Nodal Positivity in Inadequate Lymphadenectomy in Pancreaticoduodenectomy for Pancreatic Ductal Adenocarcinoma: A Population Study Using the US SEER Database
Source: Front Oncol. 2019 Dec 6;9:1386. doi: 10.3389/fonc.2019.01386 (PMC6909429; doi:10.3389/fonc.2019.01386)
Supplement: Supplementary Table 1 — The AJCC 8th TNM staging system. [file Table_1.docx]

**Supplementary Table 1. The AJCC 8th TNM Staging System**

| AJCC 8^th^ TNM staging system | | | |
| --- | --- | --- | --- |
| Ia | T1N0M0 | T1 | Tumor ≤2 cm in the greatest dimension |
| Ib | T2N0M0 | T2 | Tumor >2 cm and ≥4 cm in the greatest dimension |
| IIa | T3N0M0 | T3 | Tumor >4 cm in the greatest dimension |
| IIb | T1-3N1M0 | T4 | Tumor involves the celiac axis, the superior mesenteric artery, and/or common hepatic artery, irrespective of size |
| III | T_any_N2M0  T4N_any_M0 | N0 | No metastasis in regional lymph nodes  Metastasis in 1 to 3 regional lymph nodes |
|  |  | N1 |  |
| IV | T_any_N_any_M1 | N2 | Metastasis in≥4 regional lymph nodes |
|  |  | M0 | No distant metastasis |
|  |  | M1 | Distant metastasis |
